# Cardiac Arrhythmias Resulting from a Peripherally Inserted Central Catheter: Two Cases and a Review of the Literature

**DOI:** 10.7759/cureus.1308

**Published:** 2017-06-03

**Authors:** Jonathan Gapp, Mridula Krishnan, Felicia Ratnaraj, Robert P Schroell, Douglas Moore

**Affiliations:** 1 Internal Medicine, Creighton University Medical Center; 2 Pulmonary, Critical Care and Sleep Medicine, Creighton University Medical Center

**Keywords:** arrhythmia, bradycardia, nsvt, cardiac arrest, diabetic ketoacidosis, peripherally inserted central catheter, central venous catheter, picc

## Abstract

We present two cases of patients being treated for diabetic ketoacidosis in the intensive care unit who experienced cardiac arrhythmia secondary to peripherally inserted central catheters (PICCs). In one instance, the patient became bradycardic and experienced related loss of consciousness, ultimately requiring cardiopulmonary resuscitation. In the second case, the patient experienced an episode of nonsustained ventricular tachycardia. We explore the various types of arrhythmias that have been reported secondary to central venous catheters, as well as factors that place patients at an increased risk for arrhythmia while undergoing PICC insertion. Furthermore, we look at the literature for methods to improve the insertion of PICC lines by decreasing the risk of catheter over-insertion as well as the effects of training for PICC placement.

## Introduction

Central venous catheters (CVCs), which includes peripherally inserted central catheters (PICCs), have been shown to be associated with minor and major complications, such as arrhythmias. In a prospective trial of 1,303 patients, Yilmaziar et al. determined the incidence of arrhythmias following central line placement to be 1.6% [1]. Here, we present two critically ill patients with diabetic ketoacidosis (DKA) with PICC line placements complicated by arrhythmias.

## Case presentation

### Case 1

A 62-year-old-male with a past medical history of diabetes mellitus on oral hypoglycemic agents presented with lethargy. Vitals were a temperature of 92.1 degrees Fahrenheit (33.4 C), heart rate of 98 per minute, blood pressure of 137/91 mmHg, and a respiratory rate of 22 per minute, saturating 99% on room air. On evaluation, he was found to have venous acidity (pH) of 7.04, a partial pressure of carbon dioxide (pCO2) of 25 mmHg, bicarbonate of 7.4 mEq/L with a blood glucose of 1,304 mg/dl and an anion gap of 44. The patient was admitted to the intensive care unit (ICU) for DKA and started on an insulin drip and aggressive fluid resuscitation with the replacement of his electrolytes. A decision was made after discussion with family to place a PICC for fluid resuscitation and frequent lab draws.

**Figure 1:**
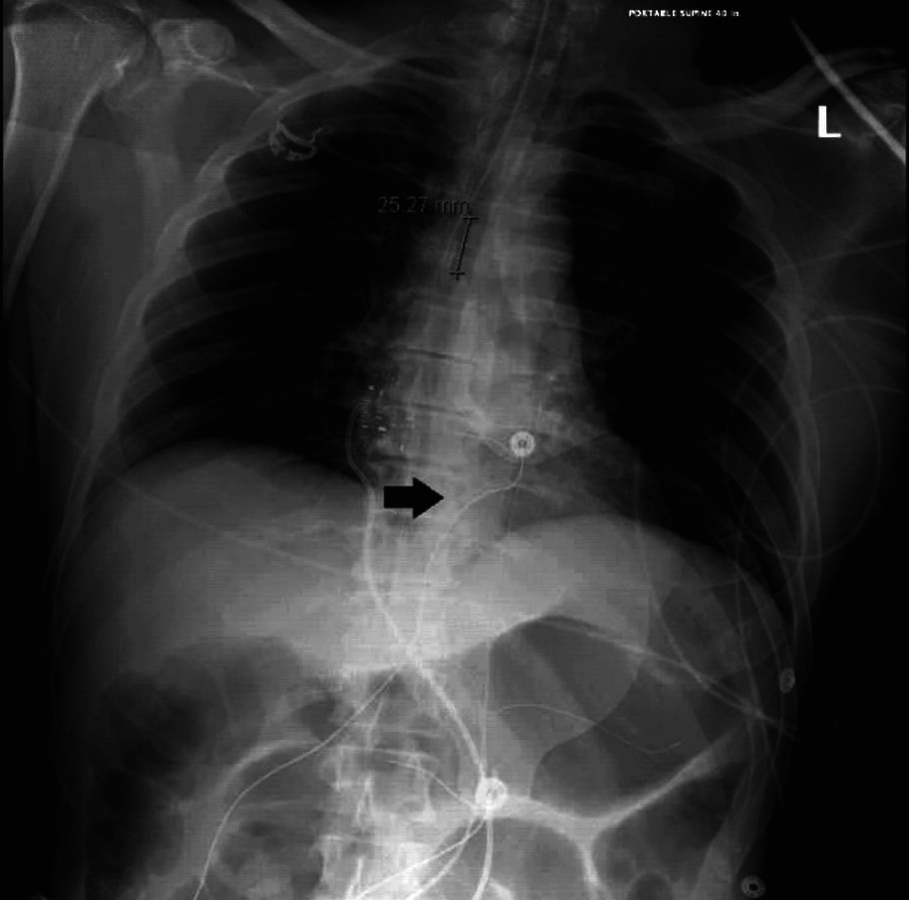
Tip of PICC (black arrow) approximately 8 cm distal to cavo-atrial junction as the likely cause of bradycardia in case 1.

PICC placement was completed by the specialized PICC nursing team. After the PICC was placed, the patient developed sinus bradycardia at a rate of 42 beats per minute per telemetry. Before atropine could be delivered, the patient became unresponsive and pulseless and a code team was called for the patient. Advanced cardiac life support (ACLS) protocol for pulseless electrical activity (PEA) was followed with cardiopulmonary resuscitation performed for two minutes and the administration of epinephrine. Resuscitation measures were successful and the return of spontaneous circulation (ROSC) was achieved. The patient was intubated and sedated. When the PICC line was flushed post-ROSC, it was noted on telemetry that the patient went into episodes of sinus tachycardia that lasted between 10-50 seconds as well as periodic wide complex ventricular tachycardia of up to 20 beats. On chest radiograph (CXR), the PICC line was noted as terminating in the right atrium (Figure 1).

### Case 2

A 23-year-old-male with a past medical history significant for Type 1 diabetes mellitus on subcutaneous insulin injections presented with four days of weakness, nausea, excessive thirst, and abdominal pain resulting from hyperglycemia due to medication noncompliance. Vitals included a temperature of 97.4 degrees Fahrenheit (36.3 C), a heart rate of 126 beats per minute, blood pressure of 175/109 mmHg, and respiratory rate of 22 per minute, saturating at 100% on room air. On evaluation, he was found to have a blood glucose of 437 mg/dl with a severe metabolic acidosis with a pH of < 6.9 on venous blood gas, a bicarbonate level of 6.0 mg/dl, and an anion gap of 30. An electrocardiographic (ECG) was obtained which showed hyperacute T waves for which he received 1 gram of calcium gluconate. Potassium was 3.8 mEq/L on admission but was initially delayed due to hemolyzed samples. The patient received 10 units of regular insulin intravenously and one ampule of bicarbonate. The patient was then admitted to the ICU for the treatment of DKA and started on an insulin drip as well as aggressive fluid resuscitation with the replacement of his electrolytes. Upon consultation of the intensive care team, a decision was made to place a PICC.

**Figure 2:**
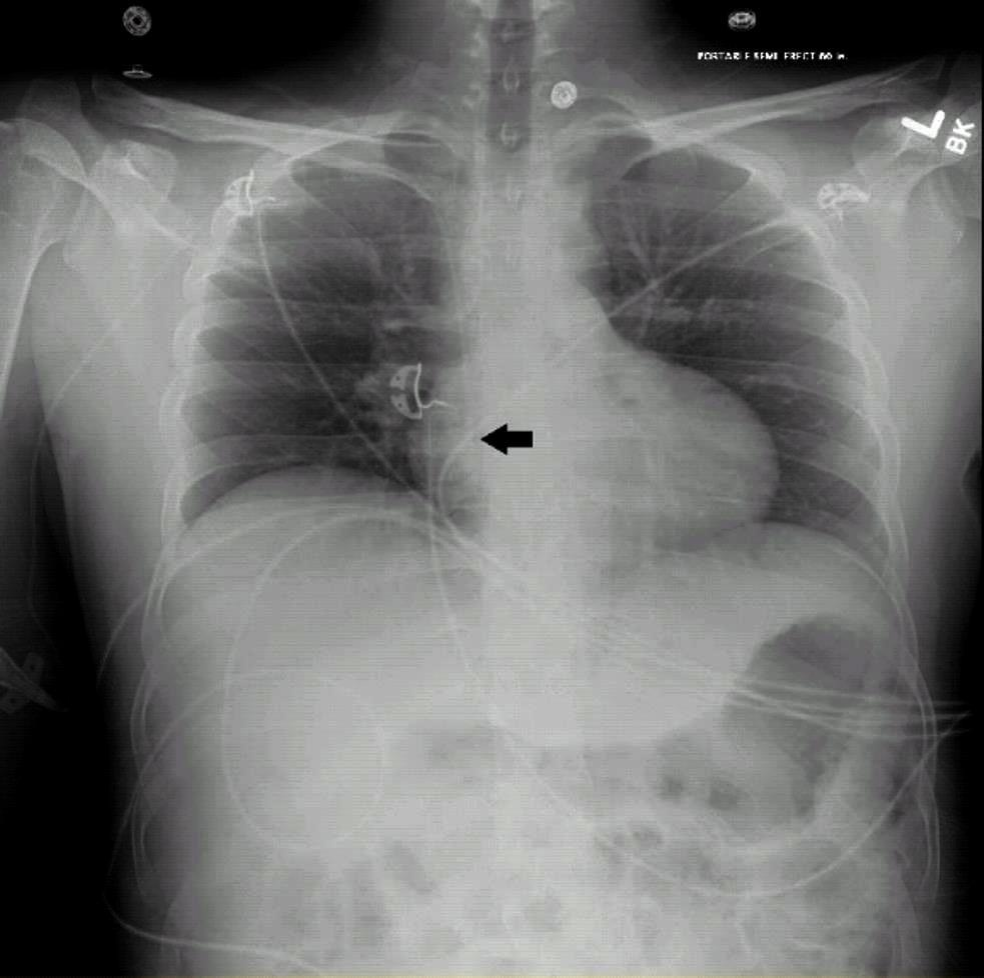
Tip of PICC in the right atrium approximately 5 cm distal to cavo-atrial junction as likely cause of NSVT in case 2.

PICC placement was performed by the specialized PICC nursing team. Immediately after placement of the PICC, telemetry showed 10 beats of nonsustained ventricular tachycardia (NSVT) with a total of 33 ventricular runs on daily telemetry, mostly between four and 10 beats. The patient remained asymptomatic; however, he received 2 grams of calcium gluconate intravenously. A CXR was obtained which showed that the distal tip of the PICC line was projecting over the right atrium (Figure 2) which was then retracted by 5 cm with a resolution of the arrhythmia. The following day, telemetry was remarkable for no ventricular runs.

## Discussion

### Introduction

In the ICU setting, cardiac arrhythmias are a common occurrence. One such etiology which must often be considered is arrhythmia secondary to CVC placement. Due to the high ratio of patients in the ICU with CVCs, this consideration becomes particularly appropriate.

In many ways, the patients presented above are similar: both are being treated for DKA and both undergoing PICC line placement with cardiac arrhythmia following shortly after. However, in one case the arrhythmia manifests as sinus bradycardia and in the other as a short run of NSVT. In both cases, the arrhythmias were attributed to over-insertion of the PICC, since both had imaging confirming over-insertion and resolution occurred following retraction of the tip of the catheter. Also interesting to note were the episodes of sinus tachycardia that occurred during saline pushes through the malpositioned catheter in the first case, contributing to our suspicion of catheter misplacement.

Most often, ventricular or atrial ectopic beats are observed in patients with CVCs [2]. However, it is important to realize that a range of cardiac arrhythmias has been documented (Table 1). Overall, sinus bradycardia (as in our first case) has been less often reported and we have found only a single other similar report by Nazinitsky, et al. [3]. As such, the incidence of bradyarrhythmias associated with CVC placements has not yet been described. Even more important to note is the occurrence of cardiac arrest following placement of the PICC line in the first case. Again, few cases have been reported of cardiac arrest associated with CVC insertion [4]. For obvious reasons of elevated morbidity and mortality of post-CPR patients, causation of cardiac arrest following PICC placement, in this case, was particularly worrisome.

**Table 1:** A Wide Array of Cardiac Arrhythmias Previously Reported as a Result of CVC Insertion CVC: central venous catheter

Author	Type of Arrhythmia
Nazinitsky, et al. (2014) [3]	Sinus arrest with bradycardia and ventricular escape rhythm
Flannery, et al. (2016) [4]	Sustained ventricular tachycardia with cardiac arrest
Chhabra, et al. (2012) [5]	Complete heart block in setting of left bundle branch block
Elsharkawy, et al. (2009) [6]	Position dependent atrial fibrillation
Yavascan, et al. (2009) [7]	Supraventricular tachycardia

In this report, we focus mainly on arrhythmias associated with PICC placement as seen in our cases here. Whenever possible, we preferred studies focusing on PICCs specifically. However, there were some instances where we felt that data on the broader class of CVCs, in general, could further inform the discussion of arrhythmias secondary to PICCs, due to similarities in catheter tip placement.

### Risk factors

Only a few studies and reports have focused on identifying underlying risk factors for developing arrhythmia secondary to CVC placement. While arrhythmias during CVC insertion have widely been attributed to mechanical irritation of the endocardium by the catheter tip, some patients may be at higher risk for arrhythmia than others.

Fiaccadori, et al. [8] studied 171 patients undergoing CVC placement and noted significantly more cases of total ventricular arrhythmias in the subgroup of patients with acute renal failure than in those with normal renal function (49% vs 15% P < 0.05). Interestingly, the same group found that there was no correlation between cardiac arrhythmia and pH derangement or previous cardiac disease in patients. Stuart, et al. [2] support this evidence in a study of 51 patients undergoing CVC placement, finding that age, cardiac history, serum potassium and catheter brand were not significant. Given these two studies, it is less likely that the arrhythmias experienced by our patients were a result of their acidosis or electrolyte imbalances associated with DKA.

In considering precipitating factors for cardiac arrhythmias in CVC placement, some have also recognized increased occurrence of complete heart block in patients with previous left bundle branch block (LBBB) as described by Chhabra, et al. [5]. The occurrence of complete heart block was suspected to be due to simple mechanical irritation by the guidewire causing transient right bundle branch block (RBBB). Normally, transient RBBB would be of no clinical significance; however, in those with pre-existing LBBB, the resultant complete heart block leads to hemodynamic instability. Therefore, previous LBBB is also a risk factor for clinically relevant cardiac arrhythmia secondary to CVC placement.

### Role of imaging post-PICC placement

The post placement CXR revealing malposition of the catheter tip in the above cases brought us to consider the role of CXR following PICC placement. While CXR did show over-insertion of the catheter, imaging following the procedure did not help us to prevent what may have been a fatal occurrence.

Use of the CXR following placement of a PICC line has long been the standard procedure to ascertain the final position of the PICC tip. Much of this has to do with its wide availability and the previous need to rule out iatrogenic pneumothorax following placement of CVCs by other approaches (ie. subclavian, internal jugular). While fluoroscopy and transesophageal echocardiogram are also highly accurate methods to confirm placement at the cavo-atrial junction, they are overly invasive and result in excessive radiation.

However, use of CXR has restrictions, such as the consistency of anatomical markers on CXR to gauge the location of the catheter tip. Furthermore, it does not allow for intraoperative surveillance of the catheter tip. This is significant in that intraoperative monitoring of the catheter tip would allow for less chance of over-insertion into the right atrium or ventricle. This would decrease the risk of a cardiac arrhythmia occurring intraoperatively or between the time that the catheter is placed and the CXR is obtained.

It is for this reason that ECG guidance of PICCs has gained popularity. In this method, the guidewire effectively becomes an intracavitary lead and can be advanced while observing the ECG tracing. The p wave morphology of the intracavitary lead is related to the position of the guidewire/lead in the superior vena cava. This allows the operator to know the position of the guidewire within the superior vena cava at that moment.

Pittiruti, et al. [9] completed a multicenter study of 1,444 patients undergoing CVC placement where 245 PICCs were placed with ECG guidance and showed 86.9% of PICC tips to be at the cavo-atrial junction and 10.2% to be in the area slightly above to 3 cm above the cavo-atrial junction. The most significant limitation of this method of PICC placement is that it cannot be used in patients with atrial fibrillation or flutter, since following the p-wave height and morphology is fundamental to the procedure.

ECG guidance was used only in the second case presented here. In that case, the follow-up CXR did display the PICC tip as over inserted, even with the use of ECG monitoring. This suggests that it may be prudent to continue the use of CXR in conjunction with ECG monitoring in order to confirm final placement of the line while still having the advantage of intraoperative guidance.

### Role of experience in line placement

The experience of the operator and the associated risk of complications has been a subject of study in the past. As would be expected, experience does significantly decrease the risk of complications in CVC placement. Sznajder, et al. [10] explored this in more depth, noting the number of complications in CVC placement to be more than twice as high in the inexperienced physician. It is worth noting, however, that the rate of misplacement of the catheter tip (as determined by CXR) was unrelated to physician experience. Since malpositioning of the catheter tip is the most likely cause of arrhythmia, this single study would suggest operator experience as being negligible as the cause of cardiac arrhythmia during CVC placement.

Vascular access teams, such as the one in place at our institution, are now common in many hospitals for placement of PICC lines. Overall, this model allows for increased training and specialization in the placement of PICC lines and will therefore likely lead to safer insertions overall.

## Conclusions

Recognizing the varied presentation and potential seriousness of cardiac arrhythmia secondary to PICC line insertion can aid in timely intervention. Furthermore, understanding measures which predispose or protect against such arrhythmias can aid in preventing these sometimes nearly lethal events. Currently, most studies performed to determine this risk are small, single center studies that are mostly quite dated. Therefore, there is a paucity of data from prospective trials to determine the cardiac risk of PICC line placement. Further implementation of available technology, such as ECG-guided PICC placement in conjunction with post-placement CXR, appears to be the safest method for decreasing the occurrence of these arrhythmias rather than using either method alone.
